# Roles of superabsorbent polymers (SAPs) and palm oil fuel ash (POFA) on the strength, cost analysis and CO_2_ emission of lightweight concrete: Comparison with aluminum-based aerated concrete

**DOI:** 10.1016/j.heliyon.2024.e40305

**Published:** 2024-11-09

**Authors:** Kittipong Kunchariyakun, Suthatip Sinyoung, Kenneth J.D. MacKenzie, Sumate Chaiprapat

**Affiliations:** a School of Engineering and Technology, Walailak University, Nakhonsithammarat, Thailand; b Center of Excellence in Sustainable Disaster Management, Walailak University, Nakhonsithammarat, Thailand; c Department of Civil and Environmental Engineering, Faculty of Engineering, Prince of Songkla University, Songkhla, Thailand; d MacDiarmid Institute for Advanced Materials and Nanotechnology, School of Chemical and Physical Science, Victoria University of Wellington, Wellington, New Zealand; e PSU Energy Systems Research Institute, Prince of Songkla University, Songkhla, Thailand

**Keywords:** Palm oil fuel ash, Lightweight concrete, Superabsorbent polymers

## Abstract

This work investigated the effects of superabsorbent polymers (SAPs) as pore-forming agent and palm oil fuel ash (POFA) as sand replacement (0–100 % by weight) on the strength, economic feasibility, and CO_2_ emissions for lightweight concrete production. The product properties were compared with the traditional aerated concrete (with aluminum powder), which aimed to shed light on the use of SAPs and POFA for manufacturing a more sustainable lightweight concrete. The use of POFA to replace sand increased the cost of production by approximately 1–7% and CO_2_ emissions by approximately 3–12 % due primarily to the transportation of the POFA from the oil palm fuel power plant, which could be avoided if produced on site of or near the power plant. The use of SAPs in the preparation of the lightweight concrete led to a reduced compressive strength compared to the aerated concrete, especially in the autoclaved samples, calculated as 15–33 % for 28 days and 44–56 % for autoclaved curing, possibly due to a collapse of the porous structure under high temperature and pressure. These drawbacks could be eliminated if the natural SAPs in the form of fine particle size were treated with Ca^2+^ in agro-waste ash so as to facilitate and enhance the pozzolanic reaction during the curing phase. The fossil-based SAPs could then be replaced with the organic-based ones, which would be a more sustainable construction material for a lower-carbon society. However, further investigations into other aspects of these materials should be conducted.

## Highlights

• Lightweight concrete made with SAPs and POFA was compared with traditional concrete.
• Strength, cost, and CO_2_ emissions of the new lightweight concrete were evaluated.
• Fast autoclave curing is less favorable for lightweight concrete with SAPs and POFA.

## 1 Introduction

Lightweight concrete has been used as a construction material for a long time due to its low density and low thermal conductivity [1,2]. The production of lightweight concrete is accomplished by introducing voids in the material matrix, which could be done by various methods, such as mixing with the lightweight aggregate or using pore-forming agents. Among different types of lightweight concrete, aerated concrete has been developed and widely used in building construction applications. The air-containing voids in aerated concrete are typically created by hydrogen gas produced in-situ by the reaction between aluminum powder and calcium hydroxide (Ca(OH)_2_) or alkalis [3,4]. Like many cementitious materials, aerated concrete is used in combination with agro-wastes such as rice husk ash (RHA) [[5], [6], [7], [8]], bagasse ash (BA) [[9], [10], [11]] and palm oil fuel ash (POFA) [[12], [13], [14], [15], [16], [17]], to reduce costs and promote waste recycling. Other benefits of utilizing agro-wastes in lightweight concrete are their low specific gravity and pozzolanic reactions of agro-wastes. Previous research has also shown that the use RHA in aerated concrete helped save energy in the autoclave curing [6]. However, incomplete burning of agro-wastes left a large amount of unburned carbon in the ash, which negatively affected the pore-forming reaction with metallic aluminum [9]. In addition, among the energy-intensive industries such as steel, cement, paper, aluminum, and plastics [18], aluminum and steel are responsible for the largest consumption of energy and, consequently, the highest degree of carbon emissions. According to statistics from the International Aluminum Institute (IAI) in 2021, the global average CO_2_ generated per kg of primary aluminum was 16.6 kg [19]. Thus, this presents a challenge to find other sustainable pore-forming materials for lightweight concrete production with ashes from agro-waste power plants.

Recently, superabsorbent polymers (SAPs) in cementitious materials have attracted increasing attention in applications, including internal curing [20,21], self-healing [22,23], and mitigating shrinkage [24]. Although the focus of these SAPs studies has been their degree of water absorption and retention [25], it was found that when the water in the SAPs particles was removed, air voids were created in the structure of a cementitious material, which resulted in the reduction of its density [26]. This property has been utilized to produce lightweight concrete. Kunchariyakun et al. [27] proposed that the lightweight concrete prepared from SAPs performs similarly to the traditional lightweight concrete. Zhang et al. [28] created millimeter-sized pores by using SAPs to produce cellular lightweight concrete and reported that the compressive and splitting tensile strengths gradually decreased with increasing porosity and pore size. Another investigation of millimeter-sized SAPs showed that the size and pore volume created by the SAPs led to a failure pattern of cellular lightweight concrete [29]. Although many workers successfully produced lightweight concrete from SAPs, there have been fewer studies of lightweight concrete prepared from SAPs and agro-waste ashes, especially in aspects of cost analysis and CO_2_ emissions. Under a global drive to reach carbon neutrality, it is essential that these factors are taken into account in the development of lightweight concrete based on SAPs and incorporating carbon based agro-waste residue.

Palm oil fuel ash (POFA) is derived from agro-waste by burning oil palm fibers in biomass power plants or boilers. Typically, POFA is disposed of in landfills, causing health, environmental, and economic problems [30]. As with other agro-wastes, POFA is a potential pozzolanic material, as it contains mainly silica. Several studies have investigated the effects of POFA in various applications and its behaviors in cementitious materials [[31], [32], [33], [34], [35], [36]]. Chinnu et al. [37] noted that the use of POFA as supplementary cementitious material improved the workability and late strength of concrete. Tangchirapat et al. [38] reported that the compressive strength of concrete improved with the additions of POFA up to 20 %. Zeyad et al. [39] reported the positive effect of POFA on the workability, strength, and permeability of concrete. Al-Shwaiter and Awang [40] showed that the inclusion of POFA in foamed concrete enhanced its fire resistance, mechanical microstructure, and transport properties [41].

To fill the gap mentioned, this present work aims to investigate the performance of lightweight concrete prepared from SAPs containing POFA, in terms of its strength, cost and CO_2_ emissions. The SAPs was used as pore-forming agent at 7 % by weight of Ordinary Portland Cement (OPC) according to the previous study [27] that yielded the concrete properties meeting the ASTM C332–17 standard [42]. According to the previous research on aerated concrete, several agro-waste ashes were utilized as silica material for sand replacement to improve its properties or production process, such as reduced autoclaving curing temperature and time [5,6]. Thus, to compare with aerated concrete, the POFA, represented as agro-waste, was utilized to replace sand at 0–100 % by weight. The results were compared and discussed with aerated concrete (traditional lightweight concrete) under both normal and autoclaved curing to investigate the potential use of lightweight concrete prepared from SAPs containing POFA. The findings of this study could contribute to the future development and preparation of low-cost sustainable lightweight concrete.

## 2 Methodology

### 2.1 Materials

**Fig. 1:**
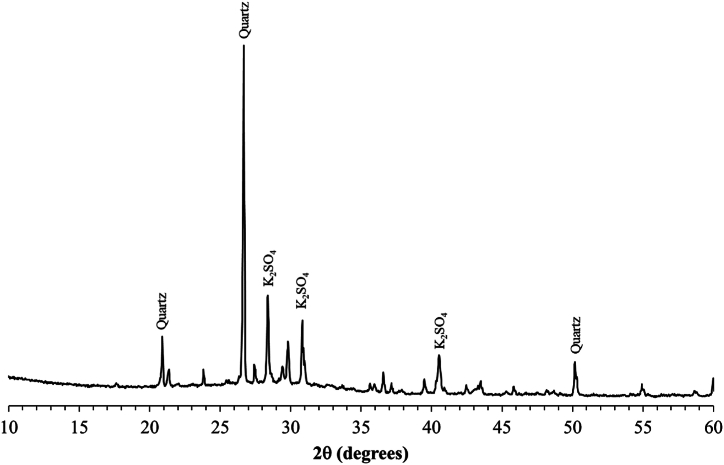
XRD pattern of POFA.

The palm oil fuel ash (POFA) used in this study was supplied by the Tha Chang Industry Group (TCG) and had an average particle size of 48 μm and a specific gravity of 2.28. Its chemical composition is given in Table 1, and its principal crystalline phases are quartz and potassium sulfate (K_2_SO_4_) (Fig. 1). The fine aggregate used in this study was river sand (S) with particle size of 150–300 μm and a specific gravity of 2.73. The binder materials were Ordinary Portland Cement (OPC) with a specific gravity of 3.14 and quick lime (calcium oxide) (L) with a specific gravity of 2.33. The chemical compositions of these compounds are shown in Table 1. The pore-forming agents used in this study were 99 % pure fine aluminum powder (Himedia) and polyacrylamide type superabsorbent polymers (SAPs) with a swelling capacity of 354 g/g.

**Table 1:** Chemical compositions of OPC, Quick lime, and POFA.

Compositions	OPC	Quick lime	POFA
CaO	69.10	93.70	12.55
SiO_2_	15.20	0.91	35.37
Al_2_O_3_	3.33	0.12	1.52
Fe_2_O_3_	3.13	0.10	2.65
Na_2_O	0.12	–	0.12
MgO	1.60	1.72	3.13
P_2_O_5_	0.12	0.01	2.89
SO_3_	3.09	–	4.41
K_2_O	0.84	–	21.86
Cl	0.03	–	2.92
TiO_2_	0.29	–	0.19
Cr_2_O_3_	–	–	0.05
MnO	0.02	–	0.17
CuO	0.03	–	0.07
ZnO	0.03	–	0.10
Rb_2_O	0.01	–	0.08
SrO	0.07	–	0.04
ZrO_2_	0.01	–	0.02
CHNO	2.99	3.44	11.86

### 2.2 Sample preparation and testing procedure

Table 2 shows the mix proportions of the samples used in this study. The mix proportions of the aerated concrete are OPC:L = 9:1, OPC:sand = 1:1, and an aluminum powder (Al) content of 0.7 wt% of the binder, designated control aerated concrete (CTA). The mix proportions of the lightweight concrete containing SAPs were OPC:sand = 1:1 and the SAPs content was 7 wt%, according to a previous study [27]. This sample was designated control lightweight concrete prepared from SAPs (CTH). The samples in which POFA was used to replace sand at levels of 25, 50, 70, and 100 wt% were designated PA25, PA50, PA75, and PA100 for the aerated concrete, and PH25, PH50, PH75, and PH100 for the lightweight concrete containing SAPs. The water contents were determined using the Flow Table method [43].

**Table 2:** Mix proportions as kg for each cubic meter.

Symbol	Mix proportion (kg)						
	OPC	L	S	POFA	Al	SAPs	Water
CTH	400	–	400	–	–	28	472
PH25	400	–	300	100	–	28	579
PH50	400	–	200	200	–	28	632
PH75	400	–	100	300	–	28	685
PH100	400	–	–	400	–	28	739
CTA	360	40	400	–	3	–	197
PA25	360	40	300	100	3	–	222
PA50	360	40	200	200	3	–	325
PA75	360	40	100	300	3	–	354
PA100	360	40	–	400	3	–	393

Remark: OPC is Ordinary Portland Cement.

L = Quick lime.

S = sand.

POFA = Palm oil fuel ash.

Al = Aluminum powder.

SAPs = Superabsorbent polymers.

The dry ingredients were mixed in Hobart mixer for 30 s before the addition of water, followed by further mixing for 1.5 min. The slurry was then poured into a 555 cm^3^ mould, stored overnight before being removed from the mould, and covered with plastic wrap until testing. Another set of specimens were cured by autoclaving at 180C for 8 h. The compressive strengths of the specimens were determined according to ASTM C109 [44] after 7, 14, 28, and 60 days and autoclaved curing. The dry density, water absorption and porosity of the samples aged for 28 days and the autoclaved samples. Prior to these physical tests, the samples were dried at a temperature of 105±5 °C for 24 h, then weighed (W_d_). The dried samples were then immersed in water for 24 h and weighed again (W_s_). The saturated samples were weighed in water (W_w_). The dry density was calculated from dry weight of the sample divided by the volume of sample. Water absorption and porosity of the samples were calculated according to Equations (1), (2)).

(1) $$Water\;absorption\;\left(\%\right)=\frac{W_{s}-W_{d}}{W_{d}}\times 100$$

(2) $$Porosity\;\left(\%\right)=\frac{W_{s}-W_{d}}{W_{s}-W_{w}}\times 100$$

### 2.3 Cost analysis and CO_2_ emission assessment

The cost analysis and CO_2_ emission assessment were conducted on the unautoclaved samples. The total cost to prepare a cubic meter of lightweight concrete was calculated taking into account the transportation cost of the raw materials and the energy cost for the manufacturing process [45] (Equation (3)).

(3) $$C_{T}=\sum_{i=1}^{n}\left(C_{MP}+C_{MT}\right)\times Q_{i}+C_{EU}$$

where *C*_*MP*_ is the cost of the raw materials (USD/kg, Table 3), *C*_*MT*_ is the material transportation cost (USD/kg), *C*_*EU*_ is the cost of the energy used in the manufacture of the lightweight concrete (USD), and *Q*_*i*_ is the amount of raw material required to produce 1 m^3^ lightweight concrete (Table 2).

**Table 3:** Cost and emission factor of raw materials for lightweight concrete for this work.

Materials	Cost^a^ (USD/kg)	Emission factor	
		kg CO_2_e/kg	Ref.
OPC	0.088	0.830	[45]
Quick lime	0.294	1.021	Ecoinvent 2.2, IPCC 2007. GWP 100a.
Sand	0.055	0.014	[46]
Al powder^b^	70.58	16.60	[19]
SAPs	9.412	3.25	[47]

Remark.

a Al powder is laboratory grade.

b Cost of materials in this work was converted to USD at a currency exchange rate of 34 Baht/dollar.

The transportation cost (*C*_*MT*_) of POFA was estimated as the cost from the Tha Chang Industry Group (TCG) to the Prince of Songkla University (PSU) where the production took place, which was estimated to be 0.10 USD/kg. The energy cost to produce the lightweight concrete (*C*_*EU*_) included the mechanical sieving of the POFA and sand and was estimated by Equation (4).

(4) $$C_{EU}=P\times t\times C$$

where *P* is the power of the machine (kW), as illustrated in Table 4, t is the time of use, and *C* is the cost of electricity used in this work as 0.12 USD/kWh.

**Table 4:** Details of equipment used in this work.

Process	Equipment	Power (kWh)
Sieving	Tyler Rx-29 Rotap	0.12
Mixing	Hobart mixer	0.6

For assessment of the CO_2_ emissions, the total CO_2_ emission (CO_2_-T) was calculated from Equation (5), which included the CO_2_ associated with the transportation, preparation, and mixing process.

(5) $$CO2-T=\sum_{i=1}^{n}Q_{i}\times F_{im}+\sum_{i=i}^{n}\frac{Q_{i}}{L_{t}}\times \frac{d}{e}\times F_{it}+\sum_{i=i}^{n}P\times t\times F_{ip}$$

where *F*_*im*_ is the emission factor of raw materials (Table 3), *F*_*it*_ is the emission factor for the energy used in this work (3 kg CO_2_/L) [48], *F*_*ip*_ is the emission factor of the power resource used in this work (0.5986 kg CO_2e_/kWh) [49], *L*_*t*_ is the material load (kg), *d* is the transport distance of POFA from the Tha Chang Industry Group (TCG) to the Prince of Songkla University (PSU) (approximately 343 km), *e* is the fuel efficiency (4.25 km/L) [45].

## 3 Results and discussion

### 3.1 Physical properties

**Fig. 2:**
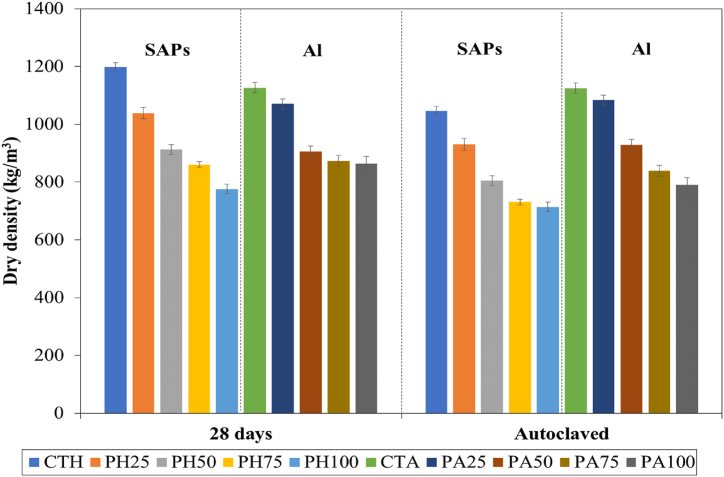
Dry density of lightweight concrete prepared from SAPs and aerated concrete under different POFA contents.

The dry density of lightweight concrete prepared from SAPs and aerated concrete with different POFA contents is shown in Fig. 2. This indicates that the replacement of sand by POFA could contribute to the loss of dry density due to the relatively low specific gravity of POFA (2.28), compared to sand (2.73). This reduction of dry density was calculated as 4–35 %, compared to the control mixtures (CTH and CTA). This result is consistent with previous studies [5,50]. Previous research has reported that the replacement of raw materials such as cement and sand by lightweight material agro-wastes clearly reduced the density of cementitious materials. Ali et al. demonstrated that the relatively light rice husk ash particles compared to cement particles caused a reduction in the density of aerated concrete [7]. Similarly, replacing BA which had lower specific gravity to sand caused a lowering density of foam concrete [51]. In terms of pore-forming agents, the dry density values of lightweight concrete prepared from SAPs exhibited similar trends to aerated concrete and were lower than autoclaved aerated concrete. When the lightweight concrete containing SAPs was cured by autoclaving, the water in SAPs particles was rapidly lost, creating pores in the sample. Under high pressure and temperature, the samples hardened and the amorphous hydration product (C-S-H, calcium silicate hydrate) crystallized. This led to almost constant growth of further C-S-H [52].

On the other hand, the SAPs particles in cementitious materials cured under traditional curing conditions [21,53] exhibited potential internal curing. Consequently, C-S-H can be formed with increasing time, decreasing the pores in the cementitious matrix and increasing the density. In the present study, the dry densities of the lightweight concrete prepared from SAPs under normal curing conditions decreased by approximately 7–15 %, under autoclaved curing.

**Fig. 3:**
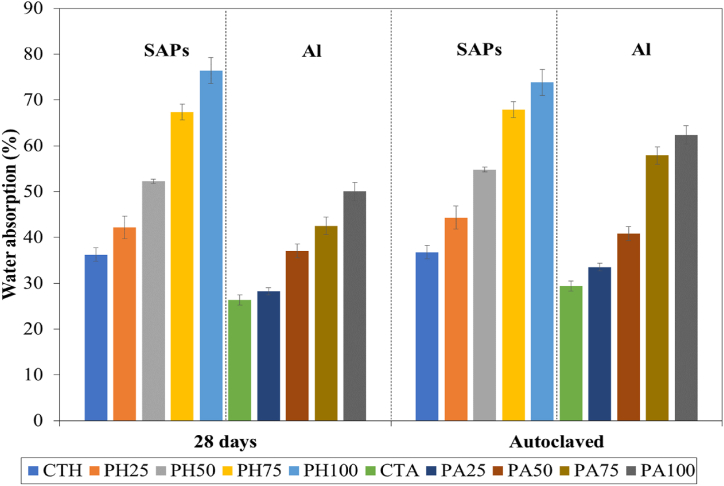
Water absorption of lightweight concrete prepared from SAPs and aerated under different POFA contents.

The water absorption of the samples was determined by their porous structure, pore volume, and density. Fig. 3 shows the water absorption of lightweight concrete prepared from SAPs and aerated concrete containing different POFA contents. These results indicate that the water absorption increases with increasing POFA content for both lightweight concrete prepared from SAPs and aerated concrete. This result is related to the dry density results. Previous research [5,51,54], also reported that lightweight concrete containing agricultural by-products, such as rice husk ash, bagasse ash, and palm oil fuel ash, could be responsible for increased water absorption due to their highly porous structure. Furthermore, the present water absorption values of lightweight concrete prepared from SAPs are found to be greater than those of aerated concrete, cured under both normal and autoclaved conditions (Fig. 3). After immersion in water, the SAPs in the lightweight concrete reabsorbed and retained the water in their particles resulting in a weight increase, by comparison with the behavior of the aerated concrete.

**Fig. 4:**
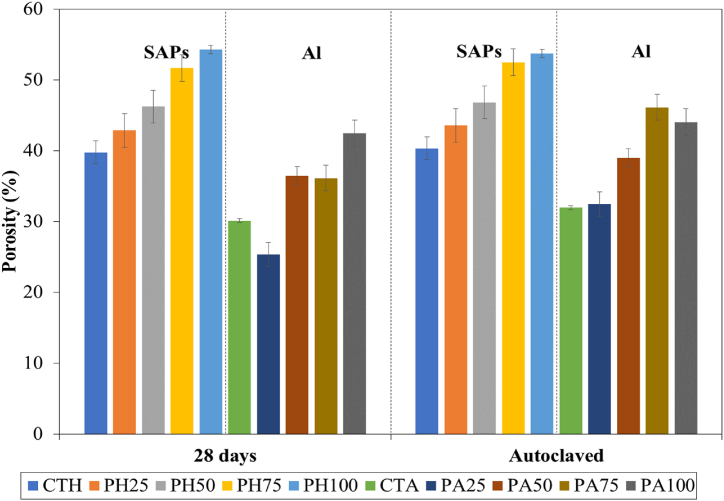
Porosity of lightweight concrete prepared from SAPs and aerated concrete under different POFA contents.

Fig. 4 shows the porosity of the lightweight concrete prepared from SAPs and aerated concrete with different POFA contents. These results indicate that the replacement of sand by POFA increased the porosity in all the samples. This result is in line with the previous study on porosity increase when the agro-waste was introduced [54,55]. Similarly, the lightweight concrete prepared from SAPs showed relatively high porosity compared to the aerated concrete. In this work, the lightweight concrete prepared from SAPs (CTH) yielded a porosity of 39.8 % and a density of 1198 kg/m^3^, which was quite consistent with the previous study on preparation of the SAP cellular concrete that had a porosity of 39.8 % and a density of 1283 kg/m^3^ [29]. This result showed that the high water uptake by the SAPs also increased the porosity, a result similar to that of the water absorption behavior (Fig. 3). The porosity of lightweight concrete prepared from SAPs was in the range of 40–53 %, while that of aerated concrete was at 31–44 %.

### 3.2 Compressive strength

**Fig. 5:**
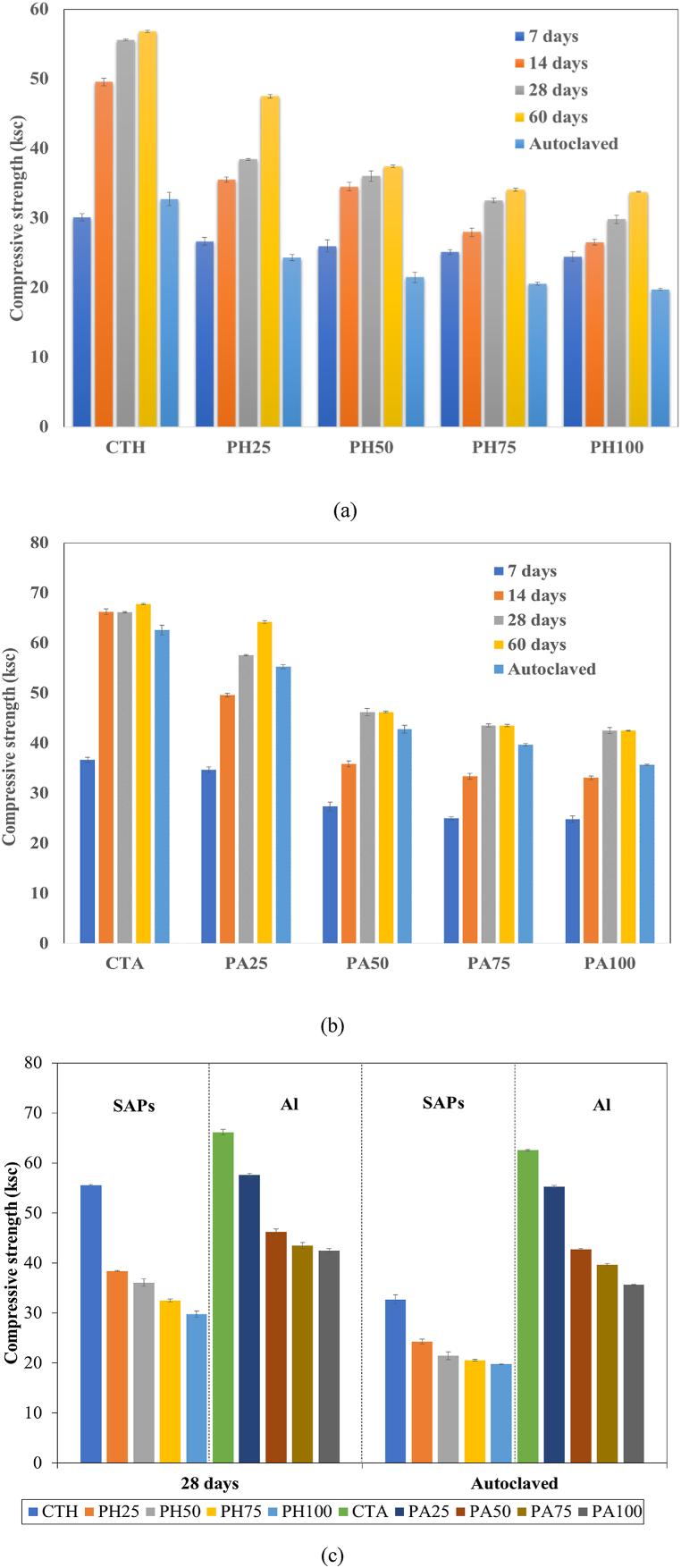
Compressive strength under different curing conditions and POFA contents. (a) lightweight concrete prepared from SAPs, (b) aerated concrete, and (c) a comparison of SAPs and Al powder.

The compressive strengths of lightweight concrete prepared from SAPs and aerated concrete with different contents of POFA are shown in Fig. 5a–c. In the aerated concrete products (Fig. 5a), the compressive strength increased with curing time but decreased with higher POFA contents. The loss of compressive strength in mixtures containing more than 20–30 % by weight of POFA has been reported in previous studies [[56], [57], [58], [59]], and has been attributed to a lack of calcium oxide for the formation of calcium silicate hydrate (C-S-H). Another possible reason for this result is the greater amount of water required for the mixtures containing POFA (Table 2), resulting in a more porous structure [5,7,9,60]. In the autoclaved samples, the compressive strength values were lower than the normal cured samples across all POFA contents. In theory, the compressive strength of autoclaved aerated concrete is predominantly derived from the formation of well-crystallized C-S-H (tobermorite), which occurs in a Si-rich environment at a Ca/Si ratio of 0.8–1.0 [52]. The presence of 21.86 % K_2_O in POFA (Table 1) reportedly contributed to the dissolution of Si ions and the formation of tobermorite [9,61]. The replacement of sand by 35.37 % of SiO_2_ in POFA had led to an increased Ca/Si ratio in the system. This setting reflected an unfavorable composition for the formation of tobermorite which explains the lower compressive strength than that of the normal cured samples at 28 days.

The compressive strength of lightweight concrete prepared from SAPs decreased with increasing POFA contents (Fig. 5b). The autoclaved samples showed relatively low strengths compared to the normal cured samples at 28 days. The ability of SAPs to retain water and release it slowly has been capitalized for use as internal curing media in various studies [20,26,53]. Mixtures containing SAPs can develop compressive strengths of 22–24 % from 7 to 28 days curing time, compared with an increase of only 18–20 % in the absence of SAPs [20]. Under autoclaved curing, the SAPs particles may collapse or be destroyed by the high temperature and pressure, which inhibits the internal curing of the SAPs. By contrast, the SAPs can contribute to compressive strength development with time in samples cured under normal conditions. According to Fig. 5b, the development of compressive strength in these samples from 7 to 28 days was 21–84 %. The internal curing effects of SAPs were clearly observed when the samples were further curing at 60 days. The strength development from 28 to 60 days of samples with SAPs was estimated to be 2–23 % for all mixtures, while there were no improvements for aerated concrete, except 2 % and 11 % for CTA and PA25, respectively. This exhibited the potential usage of SAPs and POFA for producing lightweight concrete.

Fig. 5c compares the 28-day compressive strength of lightweight concrete prepared from SAPs with aerated concrete under normal curing and autoclaved curing. Results indicate that traditional aerated concrete is of higher compressive strength than lightweight concrete prepared from SAPs at all POFA contents, especially in the autoclaved treatment. The fine-sized (0.5–3.0 mm), well-distributed pores in aerated concrete resulting from the reaction of metallic Al and alkali (Eq. (6)) [62,63], could explain the relatively high compressive strength of these samples, compared to the samples containing SAPs. As mentioned earlier, the SAPs can absorb water as much as 100 times its own weight, which enlarges the pore size upon drying. Zhange et al. (2020) [28] reported that increased pore size or porosity negatively affects compressive strength by allowing penetrating cracks to form in the concrete matrix, inducing weak spots that lead to damage. Furthermore, the mixtures containing SAPs required more water than aerated concrete to maintain their workability, consistent with compressive strength values. These effects, combined with the collapse of the SAPs particles as mentioned above, caused a considerable loss of compressive strength in the autoclaved samples.

(6) $$2Al+{3Ca\left(OH\right)}_{2}+{6H}_{2}O\;\to \;{3CaOAl}_{2}O_{3}∙{6H}_{2}O+{3H}_{2\;\left(gas\right)}$$

### 3.3 Cost analysis

In the present work, normal cured samples were selected to evaluate the cost of the lightweight products. Table 3 shows that the pore-forming agents (aluminum powder and SAPs) are the main cost factors in the production of the present lightweight concrete since these two pore-forming agents contributed to more than 70 % of the total cost of the lightweight concrete. The replacement of sand by POFA leads to an increase in the cost, taken as twice the cost of sand. This increase was due to the transportation of the POFA used in this study to our laboratory and its preparation by sieving. This led to an estimated total cost of 0.102 USD/kg, similar to that estimated by Arbelaez Perez et al. (2022) [45], who estimated the transportation and sieving cost of bagasse ash to be approximately 0.101 USD/kg. In the present case, transportation was the main cost of the POFA. No heat treatment is required for POFA in this case.

**Fig. 6:**
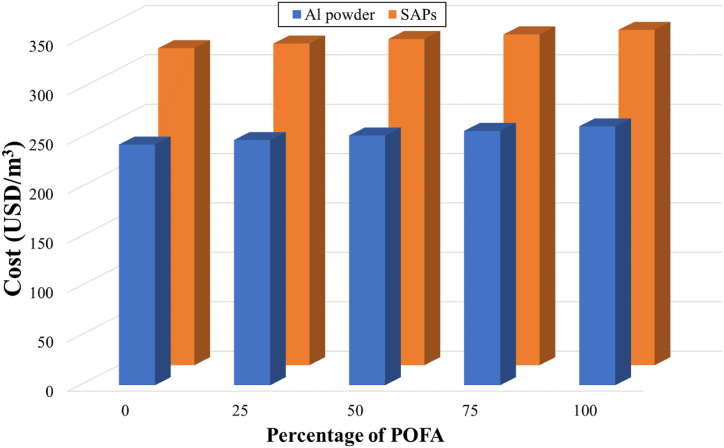
Cost analysis of lightweight concrete prepared from SAPs and aerated concrete under different POFA contents.

Fig. 6 shows that the replacement of sand by POFA increases the cost of both pore-forming agents by approximately 1–7%, compared to the control mixtures. However, these costs could be reduced if other wastes such as fly ash, bagasse ash, or banana leaf ash were substituted for cement, as shown by previous studies [45,64,65]. A comparison of the pore-forming agents showed that the mixtures containing SAPs resulted in relatively high costs compared to Al powder. The increased costs resulting from the use of SAPs to produce lightweight concrete were calculated to be 20–21 %.

However, transportation costs can be avoided if lightweight concrete is manufactured near the source of the POFA, i.e., in areas close to the power plant. Assuming a 90 % cut in POFA transportation, the production cost for the lightweight concrete would be 0.10 USD/kg, which becomes very competitive with the use of cement.

### 3.4 CO_2_ emission

**Fig. 7:**
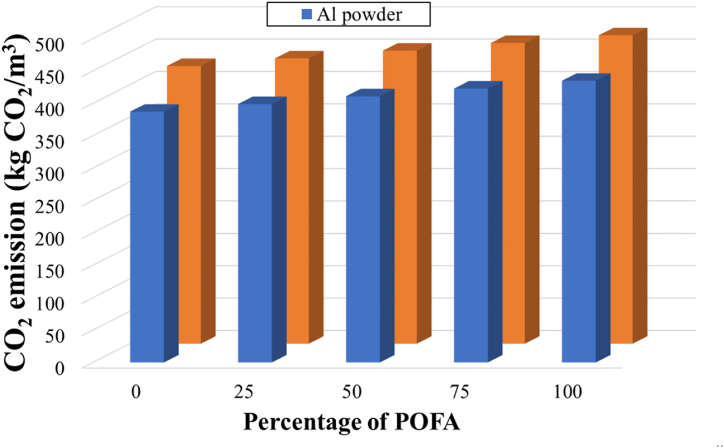
CO_2_ emission of lightweight concrete prepared from SAPs and Al-powder aerated concrete under different POFA contents.

Fig. 7 shows the CO_2_ emissions resulting from the production of lightweight concrete from SAPs and aerated concrete at different POFA contents. In the control samples, the CO_2_ emissions are 387 and 428 kg CO_2_/kg for CTA and CTH, respectively. These values agree with Lashari et al. (2023) [66], who reported the CO_2_ emission of aerated concrete to be 409 kg CO_2_/kg. The values obtained in this study are comparable with the traditional concrete, which is in a range of 365–369 kg CO_2_/kg [45,67]. Replacement of sand by POFA tends to increase the CO_2_ emissions of both lightweight concretes (Fig. 7). Based on equation (5), the CO_2_ emission of POFA was calculated as 0.133 kg CO_2_/kg, made up of 0.121 kg CO_2_/kg for transportation and 0.012 kg CO_2_/kg for the production process. This CO_2_ emission is relatively high compared to samples prepared using sand (0.014 kg CO_2_/kg). As noted in section 3.3, if the transportation distance of the POFA is reduced, the associated CO_2_ emission could be reduced by as much as 90 % CO_2_.

As was the case with the cost analysis, lightweight concrete prepared from SAPs exhibits a higher CO_2_ emission value than Al-powder aerated concrete. Although the CO_2_ emission of aluminum powder is higher than that of SAPs (Table 3), the amount of SAPs required is around 10 times that of Al powder per product unit. This gap in CO_2_ emission accounted for approximately 10 % of the aerated concrete's emission. This could open the way to produce new lightweight concrete using SAPs as the pore-forming agent combined with agro-wastes.

### 3.5 Future development of lightweight concrete

This study shows the feasibility of using SAPs for the production of lightweight concrete incorporating POFA in comparison with the traditional lightweight concrete (using aluminum powder) under both normal curing and autoclaved curing. However, aerated concrete was superior to mixtures containing SAPs in terms of cost and CO_2_ emissions. Consequently, lightweight concrete prepared from SAPs faces challenges to achieve high strength, lower cost and environmental friendliness. Nowadays, SAPs can be synthesized using a range of techniques and raw materials such as the co-polymers of acrylic acid and acrylamide, shellfish waste and chitosan [68,69]. Research on SAPs synthesis can open the door to the identification of SAPs suitable for the production of green and low-cost lightweight concrete. Natural SAPs such as alginate may be one source of sustainable SAPs for this application.

It is known that using bio-sourced superabsorbent polymers (SAPs) can significantly reduce CO_2_ emissions. For example, switching from a cationic polymer made from fossil raw materials, which has an emission factor of 3.25 kg CO_2_e/kg, to a bio-based alternative can lower emissions to 0.60 kg CO_2_e/kg [47]. Moreover, the SAP from natural alginate has the potential to be converted to the Ca^2+^ form, which could accelerate the precipitation of C-S-H by Ca^2+^ [70]. This behavior might also affect their reaction with pozzolanic materials such as rice husk ash or POFA used in the present study. A similar process in natural SAPs was reported by Thompson et al. [71], where porous composites from agar hydrogel were synthesized for use as an insulation material. In addition, it was suggested that sizes of the hydrogel had a direct impact on the properties of the porous composites, especially its mechanical properties. Many other reports have noted that the fine-size of SAPs provides better properties rather than coarse-sized materials [25,28,29,72].

Another factor that should be taken into account is the composition of the mixtures for the preparation of lightweight concrete or composites. Vafaei et al. [73] reported that a delay in the development of the solid skeleton occurred in the mixture prepared from Na_2_CO_3_ and SAPs, compared to alkali-activated slag matrices activated with NaOH or Portland cement matrices. The absorption capacity of SAPs was reportedly lowered when in contact with slurries containing Ca^2+^, as in cement paste. Thus, to achieve a successful application of SAPs for the production of high strength green, low-cost lightweight concrete, or composites containing pozzolanic materials, the properties of SAPs must address the following major challenges.

• The pore size distribution of the aerated concrete should be in the range 0.5–3.0 mm and account for 65–90 % of the total volume [62]. The size of the saturated SAPs beads for the preparation of lightweight concrete or composites should be less than 3.0 mm.
• The pozzolanic reaction requires the presence of calcium hydroxide (Ca(OH)_2_) to produce C-S-H. Natural SAPs should be modified to the Ca^2+^ form for the preparation of green and low-cost lightweight concrete or composites containing pozzolanic materials.
• The absorption capacity of the SAPs is lowered when they come into contact with a cementitious solution with a high Ca^2+^ concentration. To avoid this problem, the SAPs should be presoaked with water before mixing with the other ingredients.

## 4 Conclusion

The production of lightweight concrete incorporating POFA with SAPs could be more sustainable than the traditional one. However, the reduced dry density and compressive strength due to the low specific gravity and porous structure of the POFA made it feasible for lightweight concrete used with less load-bearing capacity applications. Cost and CO_2_ emissions from transportation played a big role in POFA to sand replacement. These could be avoided or reduced by setting this lightweight concrete production near or on the perimeter of the power plant or utilizing it as a cement replacement. Additionally, curing under normal conditions was proven preferable over the speed autoclaved curing. These drawbacks resulted from the use in this work of SAPs derived from fossil raw materials, but could be minimized or even eliminated by the use of natural pre-soaked fine-particle SAPs, modified by exchange with Ca^2+^ to enhance pozzolanic reactions with agro-wastes. Thus, the present results suggest that lightweight concretes prepared from natural Ca^2+^-exchanged SAPs and agro-wastes are interesting materials that deserve further development.

## CRediT authorship contribution statement

**Kittipong Kunchariyakun:** Writing – original draft, Methodology, Investigation, Formal analysis, Conceptualization. **Suthatip Sinyoung:** Validation, Resources, Methodology, Funding acquisition, Conceptualization. **Kenneth J.D. MacKenzie:** Writing – review & editing, Validation, Supervision. **Sumate Chaiprapat:** Supervision, Resources, Funding acquisition.

## Data and code availability

No data was used for the research described in the article.

## Declaration of competing interest

The authors declare that they have no known competing financial interests or personal relationships that could have appeared to influence the work reported in this paper.
